# Could Exposure to Glyphosate Pose a Risk to the Survival of Wild Animals? A Case Study on the Field Lizard *Podarcis siculus*

**DOI:** 10.3390/vetsci10090583

**Published:** 2023-09-21

**Authors:** Teresa Chianese, Roberta Cominale, Rosaria Scudiero, Luigi Rosati

**Affiliations:** Department of Biology, University Federico II, Via Cintia 21, 80126 Napoli, Italy; teresa.chianese2@unina.it (T.C.); r.cominale@studenti.unina.it (R.C.); luigi.rosati@unina.it (L.R.)

**Keywords:** endocrine disruptors, herbicides, liver, reproduction, reptiles, soil contamination

## Abstract

**Simple Summary:**

The use of the herbicide glyphosate in agriculture exposes wildlife to this substance. In this review, data obtained using the lizard *Podarcis siculus* as an unconventional model organism were collected and analyzed. This is to answer the question of whether occasional exposure to glyphosate can endanger the reproductive health of terrestrial vertebrates, shifting the balance of agricultural ecosystems, in which these animals play an important role by feeding on phytopathogenic organisms. The results state that glyphosate affects the liver and gonads, inducing many morphological and molecular alterations and acting as an endocrine disruptor. The data also validate the common field lizard as a valuable model organism that can provide an assessment of the toxic effect of environmental contaminants. By sharing physiological processes and reproductive mechanisms with many other animals, both aquatic and terrestrial, the information gleaned from the lizard can be transferred to other vertebrates and can serve as a starting point for the recovery of endangered wildlife.

**Abstract:**

Soil contaminants (herbicides, pesticides, and heavy metals) are among the main causes of change in terrestrial ecosystems. These substances lead to a general loss of biodiversity, both of flora and fauna and being able to biomagnify and pass through the food chain, they can endanger the survival of terrestrial vertebrates at the top of this chain. This review analyzes the risks associated with exposure to glyphosate, the active principle of many herbicide products, for the reproductive health of the field lizard (*Podarcis siculus*) potentially exposed to the substance in its natural habitat; therefore, introducing it as a possible model organism. Data demonstrate that glyphosate is toxic for this animal, affecting the health of the reproductive organs, both in males and females, and of the liver, the main detoxifying organ and closely involved in the female reproductive process. Sharing structural and functional characteristics of these organs with many other vertebrates, the information obtained with this reptile represents a wake-up call to consider when analyzing the cost/benefit ratio of glyphosate-based substances. The data clearly demonstrate that the *P. siculus* lizard can be considered a good target organism to study the reproductive risk assessment and hazards of exposure to soil contaminants on wild terrestrial vertebrates.

## 1. Introduction

Modern agriculture, with the expansion of intensive agricultural practices aimed at maximizing crop yields, provides for a wide use of pesticides and herbicides favored by the affirmation of genetically modified organisms, i.e., resistant transgenic crops. Glyphosate (N-(phosphonomethyl) glycine, Gly) and glyphosate-based herbicides (GBHs) are the world’s leading post-emergent, organophosphate, systemic, broad-spectrum, and non-selective herbicides for the control of both annual and perennial weeds [1]. As a systemic herbicide, Gly is readily translocated through the phloem to all parts of the plant, absorbed from the leaf surface into the cells, where it is translocated into the meristems of growing plants [1,2,3]. The effects of Gly are visible within two to seven days, depending on the type of weed; the primary mode of action is the blockade of the shikimate pathway, a pathway linking primary and secondary metabolism. The target enzyme of glyphosate is 5-enolpyruvylshikimate-3-phosphate synthase (EPSPS), one of seven enzymes that catalyze a series of reactions, which begins with the reaction between shikimate-3-phosphate (S3P) and phosphoenolpyruvate (PEP) and leads to the formation of the chorismate, a precursor of the biosynthesis of the aromatic amino acids phenylalanine, tryptophan, and tyrosine [1,2,3]. By inhibiting the activity of EPSPS, Gly causes a deficiency in the production of essential substances necessary for organisms that contain EPSPS to survive and propagate [3]. This pathway is absent in animals, and this explains the wide use of glyphosate in agriculture, which is considered safe for animals. However, the growing presence of Gly in the environment has attracted the attention of the scientific community regarding its actual safety against non-target organisms. In addition to the potential toxicity on animals, demonstrated by the experimental administration of glyphosate to various invertebrates and vertebrates [4,5,6,7,8], it should also be considered that herbicides can influence the environmental quality and ecosystem functioning by reducing the diversity of species, modifying community structure, food chains, energy flow patterns, nutrient recycling, as well as reducing the resilience of ecosystems [4].

The detection of glyphosate in human urine has demonstrated the absorption of this herbicide through the food chain, thus initiating a large number of studies aimed at verifying the effective lack of toxicity of glyphosate for humans and, more generally, for non-target organisms [5,6]. Experiments carried out on mammals such as mice and rats, together with data obtained on farmers occupationally exposed to Gly, have shown the risks associated with the use of glyphosate, as well as damage to various organs and systems [5,6,7,8,9,10]. Genotoxicity, neurotoxicity, reproductive toxicity in males and females, as well as teratogenicity, have also been demonstrated in these experimental models [5,6,7,8,9,10].

At the same time, a plethora of studies are investigating the effects of glyphosate on accidentally or occasionally exposed animals. In particular, research is focused on aquatic ecosystems where significant amounts of glyphosate enter by leaching, surface runoff, spray drifts, agricultural returns, and groundwater intrusions [11]. It should also be considered that glyphosate is soluble in water, where it shows a slower rate of degradation than in soil [4,12,13]. Obviously, freshwater ecosystems are the most exposed among aquatic ecosystems and studies have focused particularly on the animals occupying these ecological niches. Indeed, environmental measurements of Gly have demonstrated the presence of the active ingredient or of its metabolite AMPA in surface freshwater worldwide [4,12,13,14,15]. Among aquatic vertebrates, the toxic effects of Gly and GHBs have been extensively investigated in fish and amphibians [4,12,13,14,15]. Behavioral, morphological, and molecular changes have been associated with Gly exposure throughout the fish life cycle, from the embryonic to adult stage [15]; similar effects have also been detected in amphibians [14,16].

It is a fact that the use of pesticides in agriculture poses a risk to terrestrial biota [17,18]. However, studies have mainly focused on invertebrates such as earthworms and insects [17,18,19]; studies on terrestrial vertebrates have primarily concerned the effects of pesticides, especially following the ingestion of contaminated water and/or food [20,21]. In particular, many investigations have verified the effects of Gly on experimentally exposed laboratory mammals [22,23]. Considering wild terrestrial vertebrates, birds have generally been chosen as sentinels of environmental pollution, and many studies have investigated the effects of pesticides on adult tissues and embryos following the maternal transfer of pollutants into eggs [24,25,26]. However, birds are not models of choice for ecosystem studies due to their migratory characteristics, and for this reason, researchers focus their attention on other animals. Thus, reptiles, not generally considered for toxicological studies, are becoming good unconventional animal models. Many current studies are investigating the consequences of pesticides and herbicides on terrestrial vertebrates using reptiles as model organisms [26,27,28,29,30,31,32,33]. The use of reptiles in toxicological studies, on the one hand, avoids experimentation on mammals, reducing ethical issues; on the other hand, it allows us to evaluate the effective risk that environmental contamination poses to these animals, opening up the possibility of drawing up specific protection plans for reptiles. The first data were collected on turtles and crocodiles [34,35], while studies carried out on squamates, such as snakes and lizards, are becoming more common [26,27,28,29,30,31,32,33,36]. The latter are abundantly widespread in agricultural areas, and their diet is essentially based on insects [37]; consequently, they can be exposed to contaminants by ingestion and inhalation.

The present study summarizes data describing the toxic effects of glyphosate on the Italian lizard *Podarcis siculus*. In this review, we first describe the normal tissue conditions observed in untreated control animals and then illustrate the changes observed after Gly exposure. *P. siculus*, also known as field lizard or wall lizard, lives in agricultural environments, as well as in rural and anthropized environments such as city parks and gardens [27,28], so it can easily be exposed to Gly. Therefore, it was of particular interest to verify the effect of the active principle at the basis of numerous herbicides used both in agriculture and in the care of city green areas, on the male and female reproductive system and on the liver, the main detoxifying organ also involved in the reproductive processes of oviparous vertebrates. *P. siculus* is a seasonal breeder. The breeding season begins in late spring and ends in summer [38]. The females lay groups of 6/8 eggs in 2/3 ovulatory waves in nests on the ground or in holes. In these animals, the yolk-rich cleidoic eggs are not a fully closed system; in fact, during embryo development, they absorb water from the soil through their semi-permeable shell [39,40]. After approximately 60 days, the eggs hatch into young lizards capable of feeding. In *P. siculus*, the ovary is clustered, with the follicles resembling a bunch of grapes, similar to those of other reptiles and birds. As in all oviparous vertebrates, in the *P. siculus* female, the liver plays an essential role in reproduction, producing, under estrogenic stimulation, the phospho-lipo-glycoprotein vitellogenin (VTG), the main constituent of the yolk [41]. The testis consists of seminiferous tubules and is morphologically and functionally similar to that of all other amniotes, including mammals. In spring, the testis becomes functionally active, concurrent with follicular growth and vitellogenesis in females, leading to the production of a huge amount of mature spermatozoa [32,42].

The data collected allow for greater awareness of the risks run by these small animals, which play a key role in the agricultural ecosystem, feeding on many plant-pathogenic insects and being themselves prey to birds and little mammals. In addition, information obtained from the studies performed on this animal may increase knowledge on the toxicity of Gly in other vertebrates, including mammals.

## 2. Morphological and Functional Organization of *P. siculus* Gonads

The squamate lizard *P. siculus* is a seasonal breeder and, as such, displays different organizations of the gonads during different times of the year. The ovaries are characterized by a clustered structure, with perifollicular cells located in two germinal beds; follicles have a complex epithelium that is functional for oocyte maturation and growth [43]. The primary follicles are surrounded by a monolayer of small stem cells, each of which rapidly divides into two cells, one that maintains a stem function and remains close to the outer connective theca and another that contacts the oocyte, forming an intercellular bridge [44]. Later, the follicular cells greatly increase in size and change shape, becoming pyriform cells. Pyriform cells are nurse cells that degenerate by apoptosis before the onset of vitellogenesis [45]. At this stage, small stem cells form the steroidogenic epithelium of vitellogenic follicles. In *P. siculus*, reproduction occurs with two or three ovulatory waves in spring/summer, followed by a summer rest period and an autumn recrudescence, in which ovarian functions are partially resumed but soon arrested by the onset of winter stasis [46].

The reproductive cycle of males can be divided into six different phases, during which the testes are characterized by seminiferous tubules whose cellular composition varies: summer stasis (July–August), early (September) and mid-autumn recovery (October–November), winter stasis (December–February), spring recovery (March–April), and the breeding season (May–June) [32,42]. As in females, maximum reproductive activity occurs in spring/summer, followed by a summer rest period and an autumn resumption [47]. Stases are periods of refractory or blocked testicular activity, while resumptions are periods of sperm production. In detail, during summer stasis, the seminiferous tubules are composed of Sertoli cells, and the only germ cells are the spermatogonia; then, in early and mid-autumn recovery, when renewal of spermatogenesis occurs, the tubules are composed of spermatogonia, spermatocytes I (early autumn), spermatocytes II, spermatids, and few nonfunctional spermatozoa (mid-autumn). The morphological characteristics of the testis at mid-autumn recovery remain more or less the same in winter and early spring. During the breeding season, the tubules consist of germ cells in all stages of differentiation with numerous spermatozoa, ready to be ejaculated [42,47].

## 3. Morphological and Functional Organization of *P. siculus* Liver

The liver of *P. siculus* is a large, bilobated, dark red organ located anterior to the stomach. As in all other vertebrates, it plays a key role in the metabolism of lipids, glucose, and amino acids [48,49]. The liver takes part in the production and storage of glycogen, which serves as an energy reserve to be used in various situations, such as sexual activity during the reproductive period and metabolic changes at different periods of the year. The liver is the initial processing site for materials absorbed from intestinal capillaries and transported through tributaries of the hepatic portal vein; it also metabolizes drugs and detoxifies chemicals, making them less harmful to the body by removing them from circulation [49]. In lizards, as in all oviparous vertebrates, the liver shows a wide sexual dimorphism, which highlights the different needs between the sexes for reproduction. It is the main site of production of major oocyte constituents, such as the glycol-phospho-lipo-protein vitellogenin and eggshell proteins [41,50]. The liver of *P. siculus* shows the typical hepatic architecture consisting of cords of hepatocytes radiating from the central vein towards the periphery; the hepatic sinusoids occupy the spaces between the cords; hepatocytes show a round, well-defined nucleus containing a single nucleolus and dense, marginally vacuolated cytoplasm [51]. The sinusoids are capillaries lined by endothelial cells and macrophages, which in the liver are called Kupffer cells. Finally, aggregates of pigment-containing cells called melano-macrophages (MMs) can be observed in the hepatic parenchyma, which have various functions, such as melanin synthesis, phagocytosis, and free radical neutralization [52].

## 4. Treatments Used in Studies on the Effects of Glyphosate on *P. siculus*

Data on the effects of Gly in *P. siculus* tissues were obtained by exposing these animals to pure technical-grade Gly, as described in our previous works [53,54,55,56]. Briefly, sexually mature specimens were captured in uncontaminated wooded areas of the Campania region (Italy) and exposed orally to two different concentrations of the active ingredient (0.05 and 0.5 µg/kg body weight) three times a week for 3 weeks. The animals were fed with uncontaminated insect larvae and water ad libitum; glyphosate was administered by pipetting 50 µL of an aqueous solution containing dissolved glyphosate directly into the animal’s mouth; control animals similarly received only water.

For each Gly concentration, 10 females and 10 males were treated; similarly, 10 females and 10 males constituted the control group. Both concentrations are considered rather low because they are far lower than the concentrations of the herbicide generally used in agriculture [57]. In these experiments, it was preferred to use low but repeated doses of Gly, thus mimicking the possible accumulation that these animals can suffer by feeding on contaminated food and water, even from a single spraying of the herbicide. After the treatments, animals were killed by decapitation after deep anesthesia (ketamine hydrochloride, 325 g/kg body weight); liver and gonads slices were removed and used fresh after fixation in Bouin’s and freezing for subsequent investigations, which led to the results described in [53,54,55,56] and summarized below.

## 5. Effects of Glyphosate in *P. siculus* Females

**Figure 1:**
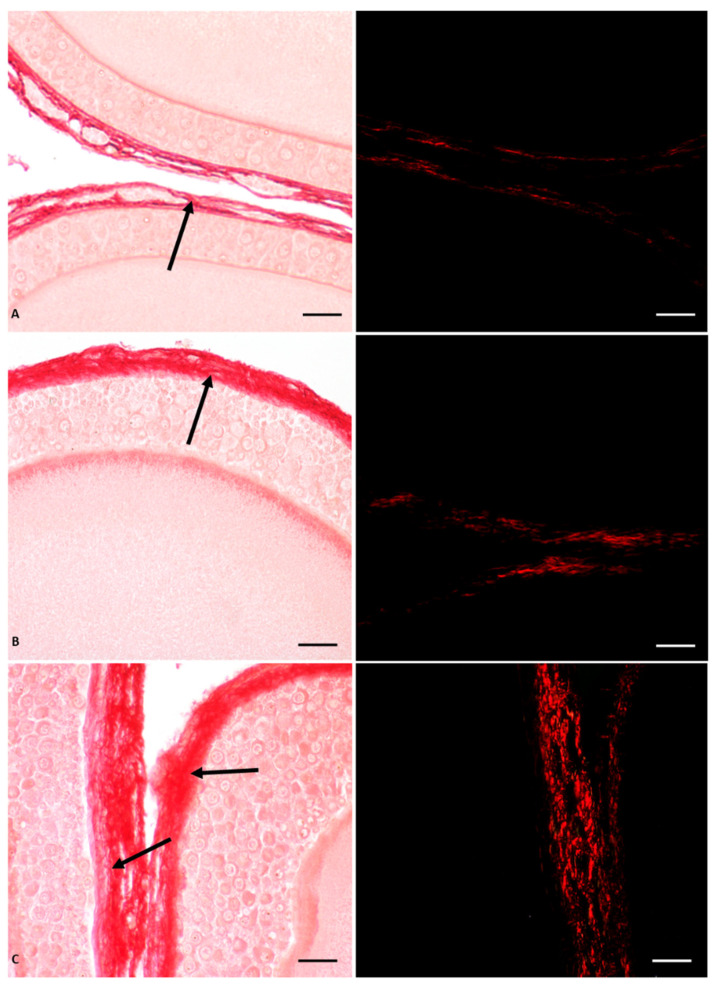
Picrosiurs red staining of *P. siculus* ovary treated with different concentrations of Gly. (**A**) Control section. (**B**) Section of animals orally exposed to a low dose of Gly (0.05 µg/kg body weight). (**C**) Section of animals orally exposed to a high dose of Gly (0.5 µg/kg body weight). The single arrow indicates the point where fibrosis occurs; collagen overdeposition (red color) was shown in the theca area of the follicle. The same sections observed with polarised light (images on the right) show the specific red spots representing fibrotic collagen deposition. The scale bars correspond to 20 µm.

In *P. siculus* females, a significant impact of Gly on ovarian function and structure was demonstrated, with a dose-dependent effect (Figure 1). In general, this herbicide induced germ cell recruitment and altered follicular anatomy by anticipating apoptotic regression of pyriform cells. In addition, it induced thecal fibrosis and affected the organization of the oocyte cytoplasm and zona pellucida (Figure 1). At the molecular level, Gly stimulated estrogen receptor synthesis through a sophisticated mechanism of endocrine interference [55].

In detail, through histological investigations, we demonstrated that the ovary of control animals contained a greater number of oocytes than the ovary of Gly-treated animals; the latter had a higher percentage of diplotene oocytes and previtellogenic follicles, thus indicating an acceleration of the oogenesis process. In addition to promoting the progression of oocyte differentiation, Gly also altered the general structure of the oocytes and the follicles; in treated animals the follicles were characterized by a modified epithelium that anticipated the apoptosis of the pyriform cells, as well as by altered interactions between the follicular cells themselves, by a disorganized oocyte cytoplasm and by a wavy vitelline envelope [55]. In parallel with the anticipation of pyriform cells apoptosis, Gly appeared to induce early activation of small cell proliferation; in fact, these cells were found to be positive for cell proliferation marker (PCNA) and were found to be arranged in many layers in the epithelium of the primary follicles. The empty spaces recorded between the pyriform cells were attributed by the authors to the action of Gly on E-cadherin, a cell junction protein very abundant in pyriform cells, which, after the treatments, changed its localization from the cell periphery to the inner cytoplasm [51]. The changes in the follicular epithelium were accompanied by alterations in the theca cells, where Gly-induced collagen hypersecretion was observed using Picrosirus red staining specific for the localization of this protein (Figure 1). We hypothesize that the excess of type III collagen observed in the perifollicular area of Gly-treated ovaries led to ovarian fibrosis, which, in turn, could hinder the ovulation process as well as the normal vascularization of the follicles due to mechanical encumbrance [55]. The structural disorganization of the ovarian follicles results in a consequent functional alteration induced by glyphosate. In fact, among the effects recorded, the alteration of the regular glucosidic composition that surrounds the follicular cells and characterizes the vitelline envelope was highlighted. In particular, in the latter, glyphosate caused in a dose-dependent manner the disappearance of fucose, a fundamental sugar involved in the fertilization process, since it is the molecule recognized by the spermatozoa when it reaches the ovulated oocyte [55]. The morphofunctional interference observed on the ovary seems to be determined by the endocrine-disrupting activity exerted by glyphosate, probably through the direct action of this herbicide on estrogen receptors (ERs). In fact, an increase in the level of both α and β ERs was found in the ovary of Gly-treated animals compared to controls; a larger increase was detected for ER β [55].

Interestingly, overexpression of ER α and β was also observed in the liver of Gly-treated animals [53,56]. Considering that the liver controls oocyte growth in oviparous vertebrates by producing vitellogenin (VTG) under estrogen signaling [58], it is clear that any impairment in this signaling can have deleterious effects on oogenesis [59]. Along with changes in estrogen receptor expression and synthesis, other Gly-induced structural and functional injuries similar to that found in the ovary have also been described in the liver; these damages were detected in the livers of both males and females [53,56].

Table 1 lists the main Gly-induced effects observed in lizard ovarian follicles.

**Figure 2:**
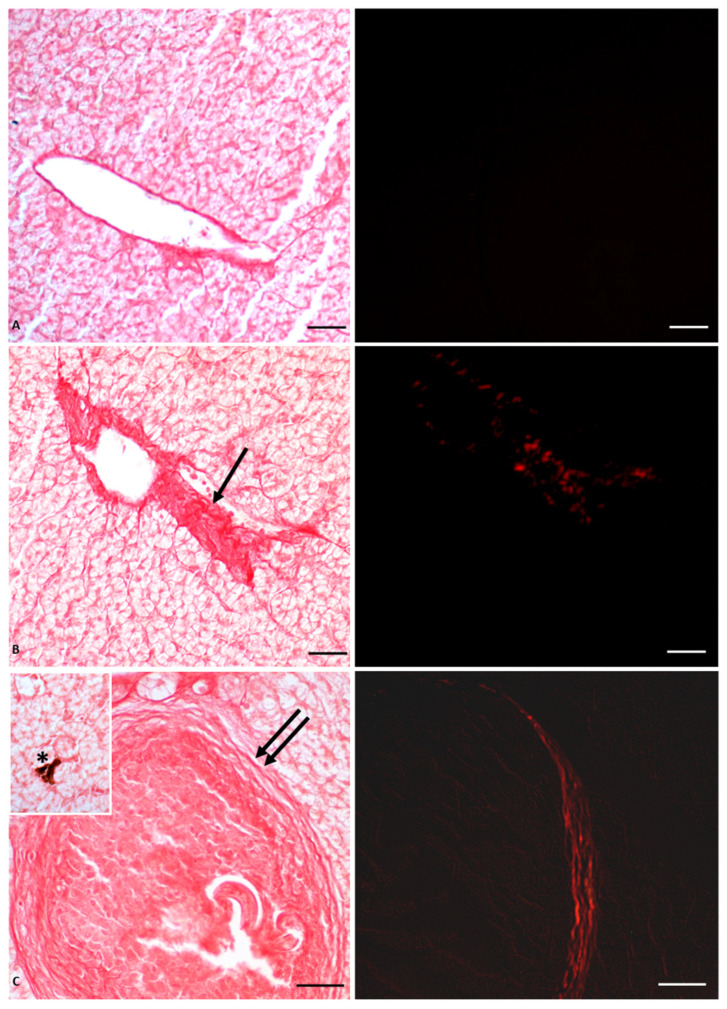
Picrosiurs red staining of *P. siculus* liver treated with different concentrations of Gly. (**A**) Control section. (**B**) Section of animals orally exposed to a low dose of Gly (0.05 µg/kg bw). (**C**) Section of animals orally exposed to a high dose of Gly (0.5 µg/kg bw). Single arrow points where fibrosis occurs; collagen overdeposition between lobules appears red stained. Double arrow indicates fibrotic nodules. Asterisk indicates a melanin granule. The same sections observed with polarized light (images on the right) show the specific red spots that represent the deposition of fibrotic collagen. Scale bars correspond to 20 µm.

Histological analysis showed massive deposition of type IV collagen, leading to the formation of large areas of fibrosis and fibrotic nodules (Figure 2); the mechanical barrier created by the collagen fiber deposits also compressed the vessels, thus altering blood fluid dynamics and inducing the observed hypertrophy of blood vessels, as shown in Figure 2. The stress condition induced by the herbicide on the liver was also manifested by the appearance of lipofuscin granules and melanomacrophages, the decrease of glycogen storage in the hepatocytes, as well as the overexpression of both enzymatic and non-enzymatic molecules involved in oxidative stress, such as superoxide dismutase 1 (Cu/Zn SOD, known as SOD1), glutathione peroxidase 1 (GPx1), metallothionein (MT), and tumor suppressor protein 53 (p53) [53,56].

Table 1 also lists the main Gly-induced effects observed in both male and female lizard livers.

## 6. Effects of Glyphosate in *P. siculus* Males

**Figure 3:**
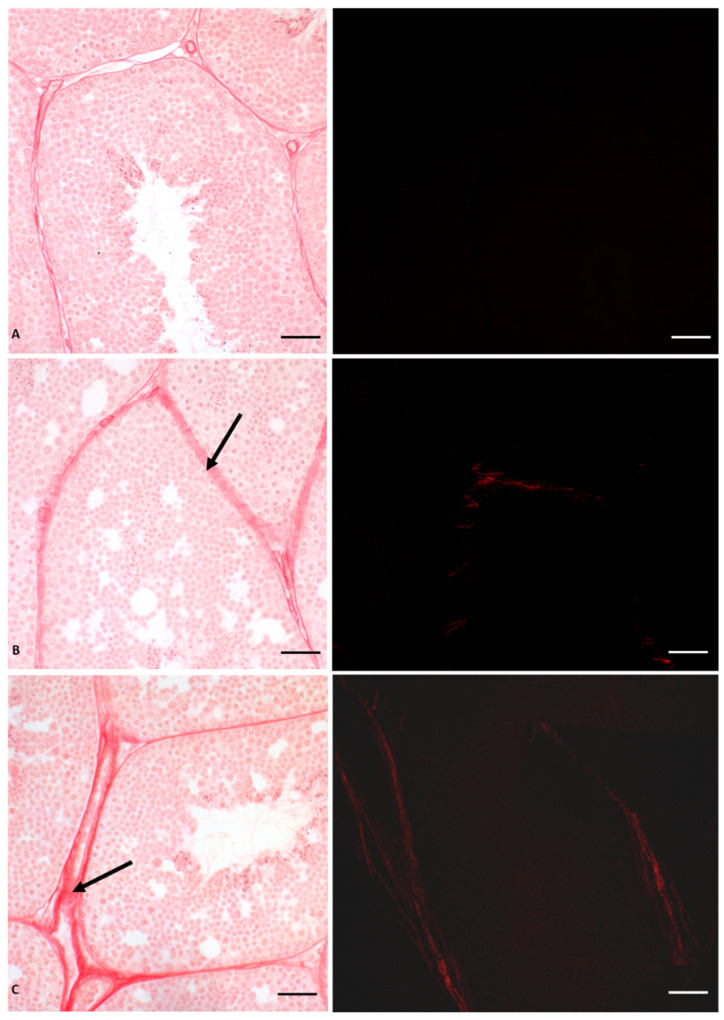
Picrosiurs red staining of *P. siculus* testis treated with different concentrations of glyphosate. (**A**) Control section. (**B**) Section of animals orally exposed to a low dose of Gly (0.05 µg/kg bw). (**C**) Section of animals orally exposed to a high dose of Gly (0.5 µg/kg bw). Arrow indicates red-stained collagen deposition between seminiferous tubules. The same sections observed with polarized light (images on the right) show the specific red spots that represent the deposition of fibrotic collagen. Scale bars correspond to 20 µm.

As mentioned above, the same lesions recorded in glyphosate-induced females, such as fibrosis, nodule formation, and altered estrogen receptor expression, were also described in the liver of *P. siculus* males [53,56]. The estrogen-like action of Gly already at a low dose was confirmed by the expression of VTG in the male liver, which is a clear sign of estrogenic contamination [60,61]. Furthermore, significant changes in *ERs* mRNA levels were also observed in the testis in a dose-dependent manner [54]. Of the two receptors, Gly mainly affects the *ERβ* expression. By real-time PCR analysis, we demonstrated that *ERβ* mRNA transcripts are more abundant than those determined for ERα in the same samples and in unexposed control males [54]. This finding was supported by immunocytochemical investigations, which showed a higher presence and wider distribution of ERs in Gly-treated testis compared to the control, with a dominant effect of Erβ [54]. These analyses also showed that Gly treatment did not affect the distribution of aromatase, a key enzyme in the conversion of testosterone into 17-β estradiol, essential for the regular course of spermatogenesis [62,63]. From these data, we conclude that Gly acts as an endocrine disruptor by targeting ERs, influencing their expression and synthesis [59]. The imbalance of the estrogen receptor system could underlie the structural changes found in the seminiferous tubules of treated animals, again with a dose-dependent effect. In fact, a reduction in the lumen of the seminiferous tubules and in the amount of spermatozoa and the alteration of the seminiferous epithelium are evident (Figure 3). In particular, we observed extensive detachment of germ cells from each other and from Sertoli cells; germ cells detached from the tubule wall tended to form aggregated structures.

We also demonstrated that the alteration of the cohesion between the cells is determined by the reduction in the treated testis of a protein responsible for cell junctions, i.e., Connexin 43, a protein particularly abundant in testis, whose expression is regulated by the activation of estrogen receptors [64]. Finally, in the interstitial area between the seminiferous tubules, a highly dose-dependent, Gly-induced deposition of type IV collagen was recorded, as reported in Figure 3. This deposition led to the formation of testicular fibrosis, as observed in the liver, regardless of sex, and in the ovary [53,54,55,56].

Fibrosis of the interstitial area could, in turn, cause the reduction in spermatozoa, as the presence of collagen could considerably limit the activity of Leydig cells located in this area, whose main function is to produce testosterone, a hormone essential for the control of spermatogenesis [42,65]. Table 1 lists the main Gly-induced effects observed in lizard testis.

## 7. Conclusions

**Figure 4:**
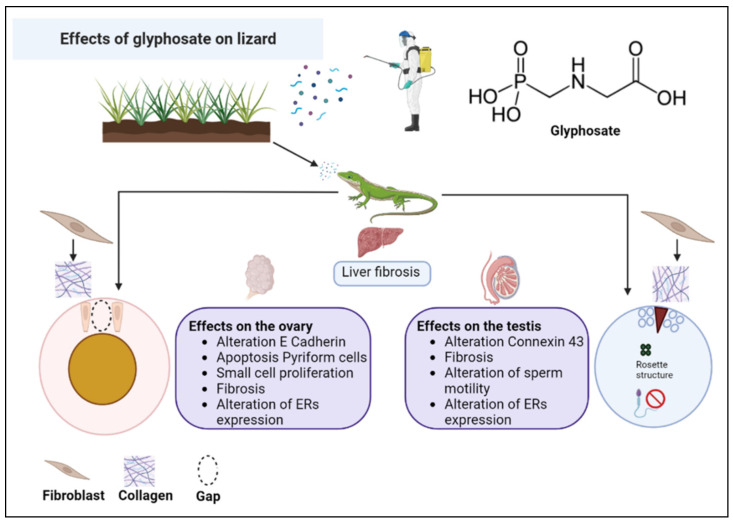
Summary scheme of the effects of glyphosate on the liver and gonads of the lizard *P. siculus*.

The results collected from our previous studies on the non-conventional model organism *P. siculus* and described in this review allow us to state that Gly seriously endangers the reproductive fitness of this lizard, causing morphological and molecular alterations in the gonads, capable of making these organs dysfunctional, probably leading to sterility over time. As illustrated in the diagram in Figure 4, the toxic effects of Gly are similar in the female and male gonad and in the liver, investigated as a detoxifying organ and involved in the female in the important process of vitellogenesis, without which there is no yolk formation, and consequently, there is no nutrient supply for the development of the embryo. The main alterations concern a general condition of cellular stress, alteration of the cell cycle, cellular differentiation and communication, changes in the carbohydrate content, and fibrosis of the connective tissues. Last but not least, Gly proves to be an endocrine disruptor, able to alter the expression of estrogen receptors in the analyzed tissues and to stimulate the estrogen-dependent expression of VTG in the male liver.

Most of these Gly-induced effects do not appear to be cell-specific, suggesting that similar effects may also be found in other cell types. This could pose an additional health hazard to animals exposed to this compound. Although some damages are dose-dependent, the cellular alterations also occur at the lowest concentration tested and for periods of exposure that are not particularly prolonged. This suggests that even accidental exposure to glyphosate can induce damage whose effects, in terms of the general and reproductive health of the organism, are difficult to quantify. One of the most important aspects that emerged from these data concerns the validation of the common field lizard *P. siculus* as a valuable model organism capable of providing an accurate assessment of the toxic effect of environmental contaminants [66].

Being an oviparous vertebrate that shares physiological processes and reproductive mechanisms with many other animals, both aquatic and terrestrial, the information gathered on this lizard can be transferred to other vertebrates and can serve as a starting point for the recovery of wildlife animals in steep decline or endangered, and therefore more difficult to study. Over the years, the lizard has adapted to multiple environmental changes, as demonstrated by its ability to live in highly anthropic environments and to occupy new geographical regions [67]. In this regard, further studies could clarify whether, even in the case of glyphosate, this animal is able to mitigate and overcome this severe stress condition, still managing to reproduce and, therefore, to survive.

## Figures and Tables

**Table 1:** Glyphosate effects recorded on *P. siculus* organs.

Liver	*Ovarian follicles*	Testis
Collagen deposition and fibrosis	Collagen deposition and fibrosis	Collagen deposition and fibrosis
Increase in glycogen, melanin, and lipofuscin granules	Changes in carbohydrate content and distribution	
	Loss of cellular adhesion	Loss of cellular junctions
Oxidative stress		
	Disorganization of theca, granulosa and zona pellucida	Impairment of spermatogenesis
	Proliferation of small stem cells and apoptosis of pyriform cells	
Endocrine disruption	Endocrine disruption	Endocrine disruption

## Data Availability

Not applicable.

## References

[1] Malik J., Barry G., Kishore G. (1989). The herbicide glyphosate. Biofactors.

[2] Duke S.O. (2018). The history and current status of glyphosate. Pest. Manag. Sci..

[3] Duke S.O. (2021). Glyphosate: Uses Other Than in Glyphosate-Resistant Crops, Mode of Action, Degradation in Plants, and Effects on Non-target Plants and Agricultural Microbes. Rev. Environ. Contam. Toxicol..

[4] Mensah P.K., Palmer C.G., Odume O.N. (2015). Ecotoxicology of Glyphosate and Glyphosate-Based Herbicides—Toxicity to Wildlife and Humans. Toxicity and Hazard of Agrochemicals.

[5] Soares D., Silva L., Duarte S., Pena A., Pereira A. (2021). Glyphosate Use, Toxicity and Occurrence in Food. Foods.

[6] Peillex C., Pelletier M. (2020). The impact and toxicity of Glyphosate and Glyphosate-based herbicides on health and immunity. J. Immunotoxicol..

[7] Bai S.H., Ogbourne S.M. (2016). Glyphosate: Environmental contamination, toxicity and potential risks to human health via food contamination. Environ. Sci. Pollut. Res. Int..

[8] Martins-Gomes C., Silva T.L., Andreani T., Silva A.M. (2022). Glyphosate vs. Glyphosate-Based Herbicides Exposure: A Review on Their Toxicity. J. Xenobiot..

[9] Costas-Ferreira C., Durán R., Faro L.R.F. (2022). Toxic Effects of Glyphosate on the Nervous System: A Systematic Review. Int. J. Mol. Sci..

[10] Marino M., Mele E., Viggiano A., Nori S.L., Meccariello R., Santoro A. (2021). Pleiotropic Outcomes of Glyphosate Exposure: From Organ Damage to Effects on Inflammation, Cancer, Reproduction and Development. Int. J. Mol. Sci..

[11] Borggaard O.K., Gimsing A.L. (2008). Fate of glyphosate in soil and the possibility of leaching to ground and surface waters: A review. Pest. Manag. Sci..

[12] Tsui M.T.K., Chu L.M. (2003). Aquatic toxicity of glyphosate-based formulations: Comparison between different organisms and the effects of environmental factors. Chemosphere.

[13] Pérez G.L., Vera M.S., Miranda L., Kortekamp A. (2011). Effects of Herbicide Glyphosate and Glyphosate-Based Formulations on Aquatic Ecosystems. Herbicides and Environment.

[14] Annett R., Habibi H.R., Hontela A. (2014). Impact of glyphosate and glyphosate-based herbicides on the freshwater environment. J. Appl. Toxicol..

[15] Lopes A.R., Moraes J.S., Martins C.M.G. (2022). Effects of the herbicide Glyphosate on fish from embryos to adults: A review addressing behavior patterns and mechanisms behind them. Aquat. Toxicol..

[16] Wagner N., Reichenbecher W., Teichmann H., Tappeser B., Lötters S. (2013). Questions concerning the potential impact of glyphosate-based herbicides on amphibians. Environ. Toxicol. Chem..

[17] Kenko D.B.N., Ngameni N.T., Awo M.E., Njikam N.A., Dzemo W.D. (2023). Does pesticide use in agriculture present a risk to the terrestrial biota?. Sci. Total Environ..

[18] Klatyik S., Simon G., Olah M., Mesnage R., Antoniou M.N., Zaller J.G., Szekacs A. (2023). Terrestrial ecotoxicity of glyphosate, its formulations, and co-formulants: Evidence from 2010–2023. Environ. Sci. Eur..

[19] Niemeyer J.C., De Santo F.B., Guerra N., Ricardo Filho A.M., Pech T.M. (2018). Do recommended doses of glyphosate-based herbicides affect soil invertebrates? Field and laboratory screening tests to risk assessment. Chemosphere.

[20] Tarazona D., Tarazona G., Tarazona J.V. (2021). A Simplified Population-Level Landscape Model Identifying Ecological Risk Drivers of Pesticide Applications, Part One: Case Study for Large Herbivorous Mammals. Int. J. Environ. Res. Public. Health.

[21] Liebing J., Völker I., Curland N., Wohlsein P., Baumgärtner W., Braune S., Runge M., Moss A., Rautenschlein S., Jung A. (2020). Health status of free-ranging ring-necked pheasant chicks (*Phasianus colchicus*) in North-Western Germany. PLoS ONE.

[22] Milesi M.M., Lorenz V., Durando M., Rossetti M.F., Varayoud J. (2021). Glyphosate Herbicide: Reproductive Outcomes and Multigenerational Effects. Front. Endocrinol..

[23] Bukowska B., Woźniak E., Sicińska P., Mokra K., Michałowicz J. (2022). Glyphosate disturbs various epigenetic processes in vitro and *in vivo*—A mini review. Sci. Total Environ..

[24] Redig P.T., Arent L.R. (2008). Raptor toxicology. Vet. Clin. North. Am. Exot. Anim. Pract..

[25] Moreau J., Rabdeau J., Badenhausser I., Giraudeau M., Sepp T., Crépin M., Gaffard A., Bretagnolle V., Monceau K. (2022). Pesticide impacts on avian species with special reference to farmland birds: A review. Environ. Monit. Assess..

[26] Liwszyc G., Larramendy M. (2023). Bird and Reptile Species in Environmental Risk Assessment Strategies.

[27] Verderame M., Limatola E., Scudiero R., Larramendy M.L. (2017). The Terrestrial Lizard *Podarcis sicula* as Experimental Model in Emerging Pollutants Evaluation. Ecotoxicology and Genotoxicology: Non-Traditional Terrestrial Models.

[28] Verderame M., Scudiero R. (2019). Health status of the lizard *Podarcis siculus* (Rafinesque-Schmaltz, 1810) subject to different anthropogenic pressures. CR Biol..

[29] Scudiero R., Motta C.M., Simoniello P. (2021). Impact of environmental stressors on gene expression in the embryo of the Italian wall lizards. Appl. Sci..

[30] Simbula G., Moltedo G., Catalano B., Martuccio G., Sebbio C., Onorati F., Stellati L., Bissattini A.M., Vignoli L. (2021). Biological responses in pesticide exposed lizards (*Podarcis siculus*). Ecotoxicology.

[31] Hoang A.Q., Tu M.B., Takahashi S., Kunisue T., Tanabe S. (2021). Snakes as bimonitors of environmental pollution: A review on organic contaminants. Sci. Total Environ..

[32] Rosati L., Chianese T., Simoniello P., Motta C.M., Scudiero R. (2022). The Italian wall lizard *Podarcis siculus* as a biological model for research in male reproductive toxicology. Int. J. Mol. Sci..

[33] Moltedo G., Catalano B., Martuccio G., Sesta G., Romanelli G., Lauria A., Berducci M.T., Parravano R., Maggi C., Simbula G. (2023). Processes involved in biochemical response to pesticides by lizard *Podarcis siculus* (Rafinesque-Schmaltz, 1810) -A field study. Toxicol. Appl. Pharmacol..

[34] Golden N.H., Rattner B.A. (2003). Ranking terrestrial vertebrate species for utility in biomonitoring and vulnerability to environmental contaminants. Rev. Environ. Contam. Toxicol..

[35] Tavalieri Y.E., Galoppo G.H., Canesini G., Luque E.H., Muñoz-de-Toro M.M. (2020). Effects of agricultural pesticides on the reproductive system of aquatic wildlife species, with crocodilians as sentinel species. Mol. Cell Endocrinol..

[36] Haskins D.L., Gogal R.M., Tuberville T.D. (2020). Snakes as novel biomarkers of mercury contamination: A review. Rev. Environ. Contam. Toxicol..

[37] Simbula G., Bissattini A.M., Vignoli L. (2022). Linking agricultural practices to lizard trophic behaviour: An ecological approach. Sci. Total Environ..

[38] Mosconi G., Carnevali O., Polzonetti A.M. (1991). Ovarian development and sex steroid hormones during the reproductive cycle of *Podarcis sicula* Raf. Gynecol. Endocrinol..

[39] Simoniello P., Motta C.M., Scudiero R., Trinchella F., Filosa S. (2011). Cadmium-induced teratogenicity in lizard embryos: Correlation with metallothionein gene expression. Comp. Biochem. Physiol. C Toxicol. Pharmacol..

[40] Simoniello P., Trinchella F., Filosa S., Scudiero R., Magnani D., Theil T., Motta C.M. (2014). Cadmium contaminated soil affects retinogenesis in lizard embryos. J. Exp. Zool. A Ecol. Genet. Physiol..

[41] Tramunt B., Montagner A., Tan N.S., Gourdy P., Rémignon H., Wahli W. (2021). Roles of Estrogens in the Healthy and Diseased Oviparous Vertebrate Liver. Metabolites.

[42] Milani L., Maurizii M.G. (2015). Vasa expression in spermatogenic cells during the reproductive cycle phases of *Podarcis sicula* (Reptilia, Lacertidae). J. Exp. Zool. B Mol. Dev. Evol..

[43] Filosa S. (1973). Biological and cytological aspects of the ovarian cycle in *Lacerta sicula* Raf. Mon. Zool. Ital..

[44] Andreuccetti P., Taddei C., Filosa S. (1978). Intercellular bridges between follicle cells and oocyte during the differentiation of follicular epithelium in Lacerta *sicula Raf*. J. Cell Sci..

[45] Motta C.M., Scanderbeg M.C., Filosa S., Andreuccetti P. (1995). Role of pyriform cells during the growth of oocytes in the lizard *Podarcis sicula*. J. Exp. Zool..

[46] Motta C.M., Tammaro S., Di Lorenzo M., Panzuto R., Verderame M., Migliaccio V., Simoniello P. (2020). Spring and Fall recrudescence in *Podarcis siculus* ovaries: A role for progesterone. Gen. Comp. Endocrinol..

[47] Della Ragione F., Comitato R., Angelini F., D’Esposito M., Cardone A. (2005). Molecular cloning and characterization of the clock gene period2 in the testis of lizard Podarcis sicula and its expression during seasonal reproductive cycle. Gene.

[48] Triger D.R. (1979). Physiological functions of the liver. Br. J. Hosp. Med..

[49] Trefts E., Gannon M., Wasserman D.H. (2017). The liver. Curr. Biol..

[50] Roy A.K., Chatterjee B. (1983). Sexual dimorphism in the liver. Annu. Rev. Physiol..

[51] Buono S., Cristiano L., D’Angelo B., Cimini A., Putti R. (2007). PPARalpha mediates the effects of the pesticide methyl thiophanate on liver of the lizard *Podarcis sicula*. Comp. Biochem. Physiol. C Toxicol. Pharmacol..

[52] Scalia M., Geremia E., Corsaro C., Santoro C., Sciuto S., Sichel G. (1988). The extracutaneous pigmentary system: Evidence for melanosynthesis in Amphibia and Reptilia liver. Comp. Biochem. Physiol..

[53] Verderame M., Scudiero R. (2019). How glyphosate impairs liver condition in the field lizard *Podarcis siculus* (Rafinesque-Schmaltz, 1810): Histological and molecular evidence. Biomed. Res. Int..

[54] Verderame M., Chianese T., Rosati L., Scudiero R. (2022). Molecular and histological effects of Glyphosate on testicular tissue of the lizard *Podarcis siculus*. Int. J. Mol. Sci..

[55] Rosati L., Chianese T., De Gregorio V., Verderame M., Raggio A., Motta C.M., Scudiero R. (2023). Glyphosate Interference in Follicular Organization in the Wall Lizard *Podarcis siculus*. Int. J. Mol. Sci..

[56] Verderame M., Chianese T., Scudiero R., Liwszyc G., Larramendy M.L. (2023). Morphological and Molecular Evidence of Active Principle Glyphosate Toxicity on the Liver of the Field Lizard *Podarcis siculus*. Bird and Reptile Species in Environmental Risk Assessment Strategies.

[57] (2015). EFSA Conclusion on the peer review of the pesticide risk assessment of the active substance glyphosate. EFSA J..

[58] Shapiro D. (1982). Steroid hormone regulation of vitellogenin gene expression. CRC Crit. Rev. Biochem..

[59] Flouriot G., Pakdel F., Valotaire Y. (1996). Transcriptional and post-transcriptional regulation of rainbow trout estrogen receptor and vitellogenin gene expression. Mol. Cell Endocrinol..

[60] Verderame M., Prisco M., Andreuccetti P., Aniello F., Limatola E. (2011). Experimentally nonylphenol-polluted diet induces the expression of silent genes VTG and ERα in the liver of male lizard *Podarcis sicula*. Environ. Pollut..

[61] Verderame M., Limatola E. (2010). Molecular identification of estrogen receptors (ERapha and ERbeta) and their differential expression during VTG synthesis in the liver of lizard *Podarcis sicula*. Gen. Comp. Endocrinol..

[62] Place A.R., Lang J., Gavasso S., Jeyasuria P. (2001). Expression of P450arom in *Malaclemys terrapin* and *Chelydra serpentina*: A tale of two sites. J. Exp. Zool..

[63] Rosati L., Falvo S., Chieffi Baccari G., Santillo A., Di Fiore M.M. (2021). The Aromatase-Estrogen System in the Testes of Non-Mammalian Vertebrates. Animals.

[64] Moinfar Z., Dambach H., Schoenebeck B., Förster E., Prochnow N., Faustmann P.M. (2016). Estradiol Receptors Regulate Differential Connexin 43 Expression in F98 and C6 Glioma Cell Lines. PLoS ONE.

[65] Zirkin B.R., Papadopoulos V. (2018). Leydig cells: Formation, function, and regulation. Biol. Reprod..

[66] Di Lorenzo M., Mileo A., Laforgia V., De Falco M., Rosati L. (2021). Alkyphenol Exposure Alters Steroidogenesis in Male Lizard *Podarcis siculus*. Animals.

[67] Oskyrko O., Sreelatha L.B., Hanke G.F., Deichsel G., Carretero M.A. (2022). Origin of introduced Italian wall lizard, *Podarcis siculus* (Rafinesque-Schmaltz, 1810) (Squamata: Lacertidae), in North America. BioInvasions Rec..

